# Maximizing Participant Engagement, Participation, and Retention in Cohort Studies Using Digital Methods: Rapid Review to Inform the Next Generation of Very Large Birth Cohorts

**DOI:** 10.2196/23499

**Published:** 2021-05-14

**Authors:** Joanna Nkyekyer, Susan A Clifford, Fiona K Mensah, Yichao Wang, Lauren Chiu, Melissa Wake

**Affiliations:** 1 Murdoch Children’s Research Institute Parkville Australia; 2 Department of Paediatrics The University of Melbourne Parkville Australia; 3 Faculty of Medicine, Nursing and Health Sciences Monash University Clayton Australia

**Keywords:** cohort studies, communication modes, digital study, mobile phone, participant engagement, research methodology, retention, systematic reviews

## Abstract

**Background:**

Many current research needs can only be addressed using very large cohorts. In such studies, traditional one-on-one phone, face-to-face, or paper-based engagement may not be feasible. The only realistic mechanism for maintaining engagement and participation at this scale is via digital methods. Given the substantial investment being made into very large birth cohort studies, evidence for optimal methods of participant engagement, participation, and retention over sustained periods without in-person contact from researchers is paramount.

**Objective:**

This study aims to provide an overview of systematic reviews and meta-analyses evaluating alternative strategies for maximizing participant engagement and retention rates in large-scale studies using digital methods.

**Methods:**

We used a rapid review method by searching PubMed and Ovid MEDLINE databases from January 2012 to December 2019. Studies evaluating at least 1 e-engagement, participation, or retention strategy were eligible. Articles were screened for relevance based on preset inclusion and exclusion criteria. The methodological quality of the included reviews was assessed using the AMSTAR-2 (Assessing the Methodological Quality of Systematic Reviews 2) measurement tool, and a narrative synthesis of the data was conducted.

**Results:**

The literature search yielded 19 eligible reviews. Overall, 63% (n=12) of these reviews reported on the effectiveness of e-engagement or participation promotion strategies. These evaluations were generally not conducted within very large observational digital cohorts. Most of the contributing reviews included multipurpose cohort studies (with both observational and interventional elements) conducted in clinical and research settings. Email or SMS text message reminders, SMS text messages or voice notifications, and incentives were the most commonly used design features to engage and retain participants. For parental outcomes, engagement-facilitation interventions influenced uptake and behavior change, including video feedback, goal setting, and intensive human facilitation and support. Participant-stated preferences for content included new knowledge, reminders, solutions, and suggestions about health issues presented in a clear, short, and personalized way. Perinatal and postpartum women valued self-monitoring and personalized feedback. Digital reminders and multiple SMS text messages were specific strategies that were found to increase adherence to medication and clinic attendance, respectively.

**Conclusions:**

This review adds to the growing literature evaluating methods to optimize engagement and participation that may apply to large-scale studies using digital methods; it is promising that most e-engagement and participation promotion strategies appear to be effective. However, these reviews canvassed relatively few strategies, suggesting that few alternative strategies have been experimentally evaluated. The reviews also revealed a dearth of experimental evidence generated within very large observational digital cohort studies, which may reflect the small number of such studies worldwide. Thus, very large studies may need to proactively build in experimental opportunities to test engagement and retention approaches to enhance the success of their own and other large digital contact studies.

## Introduction

### Background

Adult cohort studies (such as the UK Biobank, recruiting 500,000 participants and costing approximately £250 million (US $349 million) to date [1]) have demonstrated the power of mega-cohorts to transform the speed, precision, and capacity for high-value new knowledge for health and health care delivery. Unfortunately, high-profile *early life* initiatives of similar size and ambition, such as the US National Children’s Study and UK Life Study, were withdrawn despite £0.8 billion (US $1.2 billion) and £38 million (US $59 million) funding, respectively [2,3], in large part because they stumbled at the first hurdle of engagement and uptake. Others, though successful in recruitment, have had substantial attrition over time [4]. Thus, a limited science of engagement and retention poses a critical hurdle to such studies in meeting their vision of advancing human health.

Engagement is defined as “the extent to and manner in which people actively use a resource and has been operationalized as a multistage process involving the point of engagement, a period of sustained engagement, disengagement, and reengagement” [5]. Many factors may influence the engagement process at different time points. In a research study, indicators of poor engagement may include low initial uptake from the first point of contact or reduced interaction over time, in some cases leading to complete disengagement or dropout. Engagement strategies have been developed to enable cohort studies—both observational and interventional—to meet their aims (eg, improving health behaviors and outcomes) by allowing regular, sustainable engagement with large numbers of participants via remote or digital-only studies [6].

e-Engagement incorporates the participation, recruitment, and retention of participants through digital platforms. Factors that may improve participant e-engagement include its technical features, content, frequency of waves, and engagement-facilitation interventions (EFIs) [7]. User characteristics and digital platform features should also be considered. Ritterband et al [8] simplified this in their internet intervention model, hypothesizing that behavior change is influenced by the stepwise progression of environmental factors, support, and website characteristics affecting adherence, which then affect behavior change (ie, sustained participant engagement) through various mechanisms of change. Thus, maximizing e-engagement can improve the efficiency of research processes and reduce both administration costs [9] and the validity and power costs of significant and systematic nonuptake and attrition [10] in major studies.

Given the expense of longitudinal cohort studies, effective strategies that engage and retain cohort participants are critical to the integrity of research outcomes [11,12]. The retention of study participants is vital to ensure the power and internal validity of longitudinal research [13-15], whereas participant engagement is important for evaluating the efficacy and generalizability of the program under study. A review of randomized controlled trials [16] suggests that delays in participant recruitment or high dropout rates postrandomization may lead to uncertainty in treatment effectiveness and possibly confound results. For example, in the case of technology-based intervention studies, the technology may change over time if recruitment is prolonged, potentially leading to artifacts or differential effects on treatment outcomes. Proposed retention strategies involving (1) contact and scheduling methods, (2) visit characteristics, (3) study personnel, (4) nonfinancial incentives, (5) financial incentives, (6) reminders, (7) special tracking methods, (8) study description, (9) benefits of study, (10) reimbursement, (11) study identity, and (12) community involvement [17,18] may influence participant retention rates. However, there is limited experimental evidence and data for the in-depth exploration of retention strategies and their implementation.

With the recent growth in experimental research on the optimization of digital methods in longitudinal cohort research studies, there is a need for the literature to be collectively synthesized at a pace reflecting the rapid evolution of technology.

### Objective

The objective of this review is to provide an overview of strategies that enhance engagement, participation, and retention rates in large-scale digital contact studies, comparing digital methods with alternative (digital and nondigital) methods. This work has been undertaken as part of the design of the forthcoming Generation Victoria (GenV) study [19]. GenV is a whole-of-state birth and parent cohort being planned in the state of Victoria, Australia. After initial face-to-face recruitment, the majority of contact with study participants will be via digital methods. The findings of this review are expected to inform GenV and other very large birth cohort studies in planning.

## Methods

### Protocol Registration

The protocol of this rapid review was registered with PROSPERO (International Prospective Register of Systematic Reviews; registration number CRD42020155430). We followed the PRISMA (Preferred Reporting Items for Systematic Reviews and Meta-Analyses) statement to report our systematic review [20].

### Research Questions and Definitions

In the context of the administration of large-scale digital contact cohort studies, we investigated the following research questions:

1. What technical design features aid engagement, participation, and retention?
2. What EFIs aid engagement, participation, and retention?
3. What feedback is valued by parents with young children?
4. How effective are e-engagement, participation, and retention interventions?

We used the following definitions throughout:

- Engagement: the proportion of participants who receive, open, and actively engage in a survey or an assessment wave. Incorporates the study being able to contact the participant, and the participant being motivated to start the activity.
- EFI: the approach used to increase the acceptability of a web-based program.
- Participation: the proportion of participants who completed a survey or an assessment. Incorporates the participant having time to complete the activities, understanding how to complete the activities, and being willing to provide information about themselves and their family.
- Retention: the proportion of participants who participate across successive waves. Incorporates the study being able to contact the participant and the participant wanting to continue to participate.
- Review: reporting on overall findings of an included systematic review.
- Study: reporting on findings of an individual study reported within a systematic review.

### Electronic Searches

Four authors (MW, JN, SAC, and YW) developed the search strategy and refined the searches with an experienced librarian. The search queries used to retrieve our systematic reviews and meta-analyses are presented in Multimedia Appendix 1. Literature searches were performed in PubMed and Ovid MEDLINE databases using both MeSH and free-text words. The results from each search engine were downloaded into an EndNote (Clarivate Analytics) reference library and saved in Covidence (Veritas Health Innovation Ltd). Duplicate studies across the combined groups were removed. We also consulted experts and manually searched for relevant studies.

### Selection of Reviews

Two authors (selected from JN, YW, and LC) independently screened each paper title and abstract for relevance. The full text of the remaining papers was independently screened by two of the authors (selected from JN, YW, SAC, and LC). Any disagreements were resolved by consensus. To ensure a standardized process for our review, the author’s pilot-tested titles and abstracts, and full-text screening with a sample of papers. This information helped refine the inclusion and exclusion criteria.

### Inclusion Criteria

#### Study Types

As this was a rapid review, we examined only systematic reviews and meta-analyses (not individual source studies) pertinent to large-scale cohort studies. We included studies with observational and/or interventional elements conducted in clinical and research settings. As digital technology is moving so rapidly, we limited our search to reviews published between January 2012 and December 2019, reasoning that these would include relevant older studies while being most technologically relevant to the needs of cohorts being planned in the 2020s.

Studies were eligible if they evaluated at least one of the following e-engagement, participation, or retention strategies (note that testing these strategies could occur in the context of a trial of therapeutic intervention):

1. Alternative contact metrics: for example, frequency per month or year; time of the month, week, or day; duration of each contact; and reminder content and frequency.
2. Reimbursement and gifts or penalties: for example, payments for survey completion, small gifts, or store discount codes.
3. Feedback features: for example, presented as participant’s responses or performance at the point of completion; progress over time (with or without comparison with the population); a report sharable with care providers; and thank you certificates.
4. Content features: for example, assessments relevant to the life course approach or development stage of participants and/or their child; the balance of positive and negatively framed questions; ease of understanding; cognitive burden of assessment or survey items; and interest.
5. Technical and design features: for example, native or web app, can leave and return to assessment, the appearance of the interface, gamified interface, and visual progress tracker.
6. Study design features: for example, messages personalized with participant names and study staff contactable to answer questions.
7. Target participant characteristics: for example, demographic, motivation, or burden of disease.
8. Communication modes: for example, visual, auditory, text, and real person or avatar.

#### Participants

As the respondents in large birth cohorts are usually parents for the first decade of life, our primary focus was adults aged <50 years. Where evidence existed, we considered parents of children aged between 0 and 5 years.

#### Comparators

Alternative *standard* delivery strategies such as mail, fax, and other digital interventions (DIs).

#### Outcome Measures

Participant engagement in, completion of, and retention in digital study (survey and assessment) waves throughout short and long periods.

### Exclusion Criteria

For initial title and abstract screening, we excluded the following publications: the primary focus (participant) was adults ≥50 years or children as the primary respondents; publications not written in English; and publications with full text not accessible through the University of Melbourne library.

Additional criteria for the full-text review screening were not reporting our outcome metrics of engagement, participation, or retention; focusing on low-income countries; and focusing on rare or uncommon conditions such as HIV or cancer.

### Data Extraction

A data extraction template was developed and piloted by the authors (JN, YW, SAC, and LC) in 3 reviews. The template contained general review information (author and search dates), characteristics of included studies (number of relevant studies, study designs, health topics, population age, and geographic area), e-engagement, participation, and retention promoting strategies, the methodological quality of systematic reviews, and a summary of review results and conclusions.

Data were extracted independently by 2 of the authors (selected from JN, YW, SAC, and LC), and any discrepancies were resolved by consensus.

### Data Synthesis

A meta-analysis was not performed because of the heterogeneity of intervention types, study designs, study populations, and outcome variables among the included studies. Instead, a narrative summary of the findings across studies was created based on study outcomes (ie, participant engagement, participation, completion, and retention) and strategies promoting these study outcomes.

### Methodological Quality of Included Reviews

Two authors (JN and YW) independently assessed the quality of the included review methodology using AMSTAR-2 (Assessing the Methodological Quality of Systematic Reviews 2; a measurement tool to assess systematic reviews) [21]. The appraisal tool of AMSTAR-2 included 16 domains: whether there was a description of the PICO (population, intervention, control group, and outcome) components in the research questions and the inclusion criteria; the protocol of systematic review or meta-analysis; study design rationale; the literature searching strategy; study selection; data extraction; specific details of inclusion and exclusion criteria; adequate detail of the included studies; bias risk assessment of the included studies; the funding sources; appropriate statistical methods; the impact evaluation of the individual study’s risk of bias (RoB); the explanation of RoB in individual studies; a satisfactory explanation for any heterogeneity; adequate investigation of publication bias; and potential conflicts of interest. The answer options for the AMSTAR-2 were yes, partial yes, and no. *Yes* denoted a positive result. *No* represented that there was not enough information about the domain. *Partial yes* represented that it partially adhered to the standard. Discrepancies were resolved by consensus.

## Results

### Search and Screening Results

**Figure 1:**
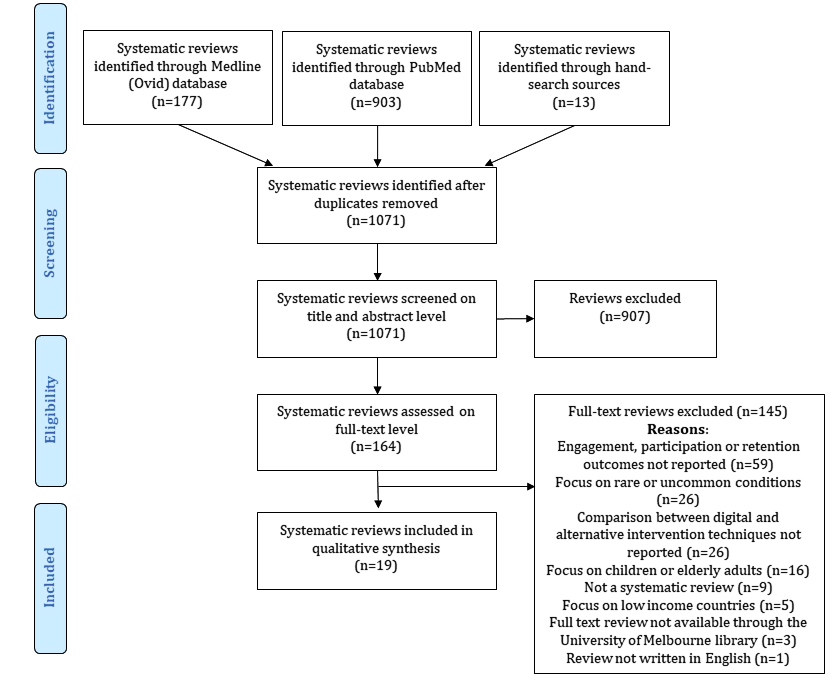
Search and screening process.

The search strategy yielded 1080 systematic reviews. An additional 13 reviews were identified through a manual search of publications’ reference lists. Following the removal of duplicates, 1071 publications were screened. The title and abstract screening excluded 907 reviews. A total of 164 articles underwent full-text review, of which 19 publications [22-40] met the inclusion criteria. Figure 1 summarizes the search and screening process presented in the PRISMA format.

### Characteristics of Included Reviews

On simple summing, the 19 reviews contained 437 studies and more than 556,000 participants (4 studies did not report the number of participants). Some studies and therefore participants are included in more than one review. Given that we aimed to identify strategies that may influence the outcomes of interest rather than synthesize an overall estimate (as per meta-analytic techniques), we did not see an overlap as problematic.

The characteristics of the included reviews are summarized in Table 1. Most reviews contained studies of varying designs, spanning quantitative and qualitative analyses.

The 19 systematic reviews included studies conducted in both research (8 reviews) and clinical or health care (11 reviews) settings. Of these reviews, 4 examined young people and adults with mental disorders, anxiety, and depressive symptoms. Others examined engagement, participation, and/or retention in digital contact studies among perinatal and postpartum women, patients with chronic diseases, parents in neonatal intensive care units, human papillomavirus vaccine uptake in young children, vaccinations in adults, pregnant women, and uptake of preschool vaccinations.

**Table 1:** Systematic review characteristics.

Study			Objective		Studies in review		Participants		Population		Regions		Designs of included studies
**Research setting**													
	Alkhaldi, 2016 [23]	Evaluate the effectiveness of tech-based prompts (eg, SMS text messages or calls) for promoting engagement with digital interventions		14		8774		Adults participating in digital interventions for physical and/or mental health		Europe, United States, and Australia		RCTs^a^	
	Baumeister, 2014 [26]	Investigate the impacts of guidance (human support) on the effectiveness of web-based mental health interventions		14		Not stated		Adults with clinical or subthreshold mental disorders		Not stated		RCTs	
	Lattie, 2019 [32]	Evaluate factors associated with the effectiveness of web-based mental health interventions		89		15,857		Postsecondary (eg, university) students targeted by universal prevention or treatment intervention programs		Europe, United States, Canada, China, Mexico, Australia, and New Zealand		RCTs, nonrandomized studies, and qualitative	
	Lim, 2019 [33]	Explore postpartum women’s and health professionals’ perspectives of digital health interventions for lifestyle management in postpartum women		9		484		Postpartum women		United Kingdom, United States, Bangladesh, and Australia		Qualitative (focus groups or interviews), questionnaire, and observational	
	Thakkar, 2016 [37]	Investigate the effect of SMS text messaging on medication adherence in chronic disease		16		2742		Adults with chronic diseases, including HIV infection, cardiovascular disease, asthma, diabetes, and epilepsy		Europe, South America, United States, Asia, and Africa		RCTs	
	Tromp, 2015 [38]	Investigate motivations of children and their parents to participate in clinical drug research		42		Not stated		Children with health conditions (eg, cancer, respiratory diseases, or diabetes) or no health conditions and their parents or guardians		Not stated		Quantitative (questionnaires or registries) and qualitative (interviews, focus groups, or case study)	
	Valimaki, 2017 [39]	Summarize the content and effectiveness of web-based interventions for depression and anxiety		27		7786		Young people (aged 10-24 years) with symptoms and/or diagnosis of depression or anxiety		Europe, United States, Canada, China, Australia, and New Zealand		RCTs	
	Whitaker, 2017 [40]	Describe the extent and effectiveness of using Facebook to recruit participants for health research		35		Median 264 per study		People (aged ≥13 years) targeted for recruitment into health studies and interventions, most commonly smoking cessation, human papillomavirus vaccination, and healthier lifestyle interventions		Germany, United States, Canada, Japan, and Australia		Quantitative and qualitative	
**Clinical or health care setting**													
	Adams, 2015 [22]	Provide evidence on the effectiveness, acceptability, economic costs, and consequences of parental financial incentives and quasi-mandatory schemes for increasing the uptake of preschool vaccinations		11		334,476		Parent of preschool children living in high-income countries; member of any relevant stakeholder group living in high-income countries		United Kingdom		RCTs and controlled pre-post and time-series analyses	
	Ames, 2019[24]	Describe clients’ experiences of receiving health information via their mobile phone		35		Not stated		Adolescent and adult clients of pregnancy, newborn, and child health, sexual health, and family planning health services receiving communication via their mobile devices		United Kingdom, United States, Canada, Southeast Asia, Australia, South America, and Africa		Qualitative study	
	Atkinson, 2019 [25]	Evaluate the effectiveness of digital push interventions in improving vaccine uptake and series completion compared with nondigital interventions		13		24,224		Adults receiving vaccines themselves, including pregnant women, or parents of adolescents and children eligible for vaccination		United States, Lebanon, Zimbabwe, and Guatemala		RCTs	
	Belisario, 2015 [27]	Compare the quality of survey responses collected using mobile apps vs other methods		14		2272		Smartphone and tablet apps as a delivery mode in clinical patients. Data collected from participants completing health-related, self-administered questionnaires		Western Europe, United States, Canada, and Korea		RCTs, crossover, and paired repeated measures studies	
	Dol, 2017 [28]	Examine the effect of eHealth interventions used in neonatal intensive care units on parents and infants		8		Not stated		Parents in neonatal intensive care units		United States, Singapore, the Netherlands, South Korea, and Israel		RCTs, quasi-experimental, pre-post, observational studies, descriptive studies, and prospective studies	
	Dubad, 2017 [29]	Evaluate the efficacy and usability of mobile mood-monitoring apps in young people		25		110,051		Healthy participants, participants from clinical populations, including youth with a range of mental health, emotional, or behavioral problems		Western Europe, United States, and Australia		RCTs, secondary analyses, nonexperimental studies, and quasi-experimental	
	Garrido, 2019 [30]	Examine the effectiveness of digital mental health interventions for depression and anxiety in young people		41		16,874		Young people with depression and anxiety		Northern Europe, United States, South America, Asia, and Australia		RCTs, single cohort (including pre-post design), and case studies	
	Kang, 2017 [31]	Evaluate the impacts of digital interventions on human papillomavirus vaccination		5		14,107		Young adults (males and females) who had received their first human papillomavirus vaccine dose		United States		RCTs	
	Mertens, 2019 [34]	Evaluate the effects of technology-supported lifestyle interventions on gestational weight gain and postnatal weight loss		9		2603		Perinatal women during pregnancy or within the first postnatal area		United States, Australia, and Iran		RCTs	
	Parsons et al, 2017 [35]	Evaluate the remotely delivered interventions for children with autism spectrum disorder living outside of urban areas: systematic review		9		197		Families having a child with autism spectrum disorder, living outside of urban areas, and having limited access to services		United States, Canada, and Australia		Pre-post, multiple-base design, RCTs, and quasi-experimental studies	
	Robotham, 2016 [36]	Assess the impact of digital notifications to improve attendance in clinics		21		16,076		Patients attending health care services		Europe, United States, Asia, Africa, and Australia		RCTs	

^a^RCT: randomized controlled trial.

### Research Question 1: Technical Design Features That Aid Engagement, Participation, and Retention

We found 4 reviews [22,25,31,32] reporting on several technical design features to aid engagement, participation, and retention, including financial incentives (including gifts), digital pushes (SMS text message alerts), voice notifications, and email or SMS text message reminders and studies’ technical feasibility and usability (ie, informed consent). Email or SMS text message reminders and SMS text message notifications were reported in 4 reviews [31,32,36,37] as the most commonly used technical design feature to improve participation and completion rates. Two reviews reported the use of email or SMS text message reminders and voice notifications [36,37] to enhance study participation. Robotham et al [36] compared zero, one, and multiple SMS text message notifications and voice notifications.

### Research Question 2: EFIs That Aid Engagement, Participation, and Retention

The following EFIs were reported by 5 reviews [24,26,28,30,35] as a means to aid uptake and participant engagement.

#### SMS Text Messages and Interactive Voice Response Messages

The review by Ames et al [24] examined perceptions and experiences of digital targeted client communication (ie, SMS text messages and interactive voice response messages) via mobile devices in the areas of reproductive, maternal, newborn, and adolescent health. The results suggested that many clients liked receiving messages from health services using mobile phones. Content preferences included new knowledge, reminders, solutions, and suggestions about health issues presented in a clear, short, and personalized way.

#### Intensive Guidance (Web-Based Interventions With Human Facilitation, Support, or Coaches)

According to the review by Baumeister et al [26], in treating mental health disorders, guidance, as a retention strategy, improved rates of completion (pooled completer rate: odds ratio 2.76, 95% CI 1.68-4.53; n=6) and the number of completed modules (pooled mean number of completed modules: standardized mean difference 0.52, 95% CI 0.37-0.067; n=7). Lim et al [33] reported on the use of lifestyle coaching as an EFI to aid DI uptake. DIs were perceived as positive, user-friendly, and acceptable. Engagement strategies employed in DIs were monitoring and feedback, goal setting, health professional input, and social support.

#### Videoconferencing and Video-Feedback Interventions

The review by Dol et al [28] reported the following EFIs to aid uptake across included studies: Baby CareLink (an educational and emotional support system for parents with children in the neonatal intensive care unit) [41], Skype, and FaceTime. In this review, no significant differences were found between parents who participated in an e-intervention or received standard care in terms of their reported anxiety and/or stress, possibly because of the greatly varied study design and type of eHealth technology across studies.

Garrido et al [30] summarized the use of web-based modules, learning materials, or activities; group chats or courses; online forums; web-based chat facilities with a mental health professional; games; and psychoeducational computer programs as EFIs to aid participant uptake. The pooled effect size on depression compared with a nonintervention control was small (Cohen *d*=0.33; 95% CI 0.11-0.55), whereas the pooled effect size of studies comparing an intervention group with an active control showed no significant differences (Cohen *d*=0.14; 95% CI −0.04 to 0.31). In addition, pooled effect sizes were higher when supervision was involved (for studies comparing digital mental health interventions with high human interaction vs no intervention: Cohen *d*=0.52, 95% CI 0.23-0.80; for studies comparing digital mental health interventions with high human interaction vs active controls with no supervision: Cohen *d*=0.49; 95% CI −0.11 to 1.01).

#### Web-Based Training Intervention in Behavioral Interventions and Video Training Materials

Parsons et al [35] reported that using video training materials compared with face-to-face training improved parent knowledge, parent intervention fidelity, social behavior, and communication skills of children with autism spectrum disorders.

### Research Question 3: Feedback Valued by Participants With Younger Children

Feedback was valued by perinatal and postpartum women and parents in neonatal intensive care units, as reported by 4 reviews [24,28,33,34].

In the review by Merten et al [34], participants valued visual and personalized feedback, information, and tools for physical activity and dietary intake tailored to their self-monitored data during pregnancy. This feedback reinforced successes and/or offered motivational support and recommendations to achieve their goals. Ames et al [24] reported that the opportunity to offer feedback about needs, preferences, and experiences during pregnancy helped develop or improve the study intervention. In the review by Dol et al [28], parents valued a video-feedback intervention that guided them to reflect on their own successful interactions through recordings of parent-infant interaction and feedback from a video interaction guidance professional. According to Lim et al [33], many of the characteristics of DIs that postpartum women valued included feedback and goal setting. Women valued setting realistic goals through video consultation with their dietitian and tracking daily weight, exercise, and blood glucose levels in a web-based intervention, consistent with known key strategies for behavior change.

### Research Question 4: Effectiveness of Engagement, Participation, and Retention Promotion Strategies

Overall, 63% (12/19) of reviews reported the effectiveness of e-engagement or participation promotion strategies. Table 2 summarizes the effectiveness of the various e-engagement, participation, and retention strategies reported in the reviews. Most findings were reported as relative rather than absolute differences, where numerical syntheses were provided (refer to Multimedia Appendix 2 [22,23,25,27,29,31,32,34,36,37,39,40] for further details on the answered research questions and strategies).

**Table 2:** Interventions, main outcomes, and results of included systematic reviews.

Study	Condition or sample	Intervention vs control	Outcome	Study statistics: number of studies, effect size (95% CI), heterogeneity	Results
Alkhaldi, 2016 [23]	Digital interventions	Study design features: (1) Technology-based engagement strategies (email, phone call, and SMS text messages) to promote engagement with digital interventions vs no strategy (11 studies); postal mail strategy (1 study); fewer technology-based strategies than the intervention group (2 studies).	Engagement—engagement with the digital intervention. Dichotomous outcomes: number of log-ins or visits, page views, sessions completed, digital interventions features used. Continuous outcomes: time spent on the digital intervention.	(1) Dichotomous outcomes (n=8): RR^a^=1.27 (−1.01 to 1.60) favoring strategy group; I^2^=71%.^b^ Continuous outcomes (n=4): SMD^c^ 0.19 (−0.11 to 0.48) favoring strategy group; I^2^=20%.	(1) Engagement in a digital intervention was higher with engagement strategy, compared with no strategy.
Baumeister, 2014 [26]	Mental health disorders	Study design features: (1) Guided interventions (with human support) vs nonguided interventions (self-guided) (2) Guided interventions with a higher qualified e-coach vs guided interventions with a lesser qualified e-coach (3) Intensive guidance (at least three email conversations per week) vs less-intensive guidance (one email contact per week).	Completion—number of completed modules, number of people completing the intervention.	(1) Number of completed modules (n=7): SMD 0.53 (0.37 to 0.67), higher in a guided group. Number of completers (n=6): OR^d^ 2.76 (1.68 to 4.53). Higher for a guided group; I^2^=42%. (2) Number of completed modules (n=4): SMD −0.15 (−0.36 to 0.05). No significant difference between groups; I^2^=0%. Number of completers (n=4): OR 0.85 (0.54 to 1.35). No significant difference between groups; I^2^=0%. (3) Completer rate: OR 1.40 (0.41 to 4.71). Higher in intensive guidance group. Mean completed modules: SMD 0.11 (−0.41 to 0.63). Higher in intensive guidance group.	(1) Completion was higher in guided interventions than self-guided interventions. (2) No difference in completion by coach qualification level. (3) Completion was higher with intensive guidance than less-intensive guidance.
Garrido, 2019 [30]	Internet or web-based interventions	Communication modes: (1) Web-based module learning materials or activities, group chats or courses, online forums, and web-based chat facilities with a mental health professional vs face-to-face counseling (2) Computer-based programs including games and psychoeducational computer programs vs waitlist control group.	Completion—proportion of commencing participants who completed the intervention.	(1) Not stated.	(1) and (2) Engagement and adherence rates were low with participants completing less than half of the intervention components.
Parsons, 2017 [35]	Internet or web-based parent training programs for autism spectrum disorder	Communication modes: (1) Web-based training intervention in behavioral interventions vs written training materials (2) Video training materials vs completing the same training face-to-face within families’ homes.	Completion—completion and adherence.	(1) and (2) Not stated.	(1) and (2) Interventions delivered via videos were more effective and accepted by parents than those delivered via written information.
Mertens, 2019 [34]	Telehealth	Communication modes: (1) Mobile apps, SMS text messages, and e-intervention vs standard care including brief information brochures with healthy eating and physical activity advice.	Participation—efficacy, feasibility, acceptability, use of e-intervention.	(1) Not stated.	(1) Email, app alerts, or SMS text message notifications are well accepted for health interventions.
Whitaker, 2017 [40]	Internet or web-based interventions	Communication modes: (1) Recruitment via Facebook advertisements vs recruitment via traditional methods or national data.	Engagement—number of participants recruited, conversion rate.	(1) Not stated.	(1) Facebook can be successfully used to recruit young and hard-to-reach populations. Facebook-recruited samples were generally representative to the target demographic, but some reported overrepresentation of young White women.
Atkinson, 2019 [25]	Vaccinations	Communication modes: (1) Digital push notifications (eg, SMS text message alerts) vs nondigital interventions (eg, appointment card). (2) Digital push notifications (eg, SMS text message alerts) vs nondigital pull interventions	Participation—vaccination uptake (1 dose) or completion (all doses in series).	(1) 1 dose (n=9): OR 1.17 (1.10 to 1.23); I^2^=89%. Completion of all doses (n=4): OR 1.53 (1.13 to 2.08); I^2^=82%. (2) 1 dose or completion of all doses (n=10): OR 1.22 (1.15 to 1.30); I^2^=79%.	(1) and (2) There were increased odds of participants being vaccinated or completing the vaccination series with digital alerts compared with nondigital interventions.
Dubad, 2018 [29]	Delivery mode	Communication modes: (1) Mobile mood-monitoring apps vs paper diary or in person.	Participation—completion rate of diary entries and mood assessments, engagement with the app.	(1) Not stated.	(1) Participation rates ranged between 30% and 99%.
Belisario, 2015 [27]	Delivery mode	Communication modes: (1) Smartphone app questionnaire vs paper questionnaire.	Completion—data completeness.	(1) Not stated.	(1) Higher data completeness in app than paper reported by individual studies.
Dol et al, 2017 [28]	eHealth intervention	Communication modes: (1) Videoconferencing (Skype or FaceTime), Baby CareLink (an internet-based application), video-feedback intervention, and internet-based telemedicine program vs standard care.	Completion—parents completed demographic and feasibility surveys postintervention.	(1) Not stated.	(1) Parents generally found eHealth interventions useful and acceptable for neonatal intensive unit care for their infant.
Valimaki, 2017 [39]	Internet or web-based interventions	Communication modes: (1) Web-based interventions for depression and anxiety, computers, tablets, or mobile phones vs waitlist, other intervention method or program.	Completion—attrition, number of participants leaving the study early.	(1) Attrition of web-based interventions compared with a control group for short-term effectiveness (n=11): OR 1.31 (1.08 to 1.58). Attrition in midterm (follow-up measurements after 3-5 months) effectiveness (n=3): OR 1.65 (1.09 to 2.49).	(1) Adolescents in the intervention group left the study early more often, both in short-term studies and midterm studies.
Robotham, 2016 [36]	Patients attending various health care services	Contact metrics: (1) One SMS text message notification vs no SMS text message notifications. (2) 2+SMS text message notifications vs no SMS text message notification. (3) SMS text message notifications vs voice notifications.	Participation—attendance, cancellation, and “no shows” at a health care service appointment.	(1) Attendance (n=13): RR 1.23 (1.10 to 1.38) in favor of the SMS text message group; I^2^=82%. Cancellation (n=3): RR 1.37 (*P*=.34) with no difference between groups; I^2^<1%. “No shows” (n=16): RR 0.75 (0.68 to 0.82); I^2^=21%. (2) Attendance (n=13): RR 1.49 (1.17 to 1.88) in favor of 2+notifications group; I^2^=66%. 19% risk difference “No shows”: (n=15): RR 0.75 (0.57 to 0.99) with “no shows” lower in the 2+notifications group or I^2^=35%. 0.3% risk difference between 1 and 2+notification groups. (3) Attendance (n=3): RR 0.90 (0.82 to 0.98) in favor of voice notifications, I^2^<1%; “No shows” (n=4): RR 1.12 (0.90 to 1.38), I^2^=73% Between 1 and 2+notification groups. “No shows”: (n=15): RR 0.75 (1.17 to 1.88) with “no shows” lower in the 2+notifications group; I^2^=35%. 0.3% risk difference between 1 and 2+notification groups. Attendance (n=3): RR 0.90 (0.82 to 0.98) in favor of voice notifications, I^2^<1%. “No shows” (n=4): RR 1.12 (0.90 to 1.38); I^2^=73%.	(1) Patients who received SMS text message notifications were 23% more likely to attend, equally likely to cancel, and less likely to *no show* a clinic appointment than those who received no notification. (2) Participants who receive 2+SMS text message notifications are 19% more likely to attend compared with one SMS text message notification but equally likely to *not show* at a clinic appointment, compared with those who received 1 SMS text message. (3) Voice notifications may increase clinic attendance slightly compared to SMS notifications, but no difference was found for “no shows”
Thakkar, 2016 [37]	Various chronic conditions	Contact metrics: (1) SMS text message reminder vs no SMS text message reminder.	Participation—medication adherence.	(1) Adherence to medication schedule (n=not stated): OR 2.11 (1.52 to 2.93). Weighted mean effect size (n=not stated): Cohen d=0.41 (0.23 to 0.59).	(1) The odds of medication adherence more than doubled with SMS text message reminders, compared with no reminders. Assuming baseline medication adherence was 50%, this translates to an improvement to 67.8%, or an absolute increase of 17.8%.
Lattie, 2019 [32]	Participation reminders	Contact metrics: (1) Email reminders vs no email reminders.	Completion—number of sessions or assessments or prompts completed.	(1) Number of sessions completed (n=not reported): Reminder group mean 2.9, SD 2.5; no reminder group mean 3.6, SD 2.3; t=0.88.	(1) Email reminders were not associated with completing more sessions in a web-based intervention. There were also notable rates of participant attrition and early program discontinuation in many of the studies.
Kang, 2017 [31]	Reminders	Contact metrics: (1) 7 email or SMS text message reminders vs paper appointment card.	Completion—completion of three-dose human papilloma virus vaccine schedule.	(1) Number of people completing the vaccine schedule (n=86): email or SMS text message reminder group 34%, paper card reminder group 32%; *P*=.76.	(1) Completion rates of a vaccine schedule did not differ by reminder format (email or SMS text message, compared with paper card).
Adams, 2015 [22]	Incentives	Reimbursement and gifts or penalties: (1) Cash lottery tickets for attendance vs usual care (no incentives). (2) Loss of US $40 welfare benefits for not vaccinating vs usual care (no incentives). (3) Loss of some welfare benefits for not vaccinating vs usual care (no incentives).	Engagement or uptake—uptake of preschool vaccinations; up to date with 0-2–year vaccinations; up to date with child vaccinations.	(1) At each follow-up time point, attendance for any reason and for vaccination was higher in incentives group. (2) No difference in up-to-date rates at 1 or 2-years follow-up. (3) Welfare deduction group had higher vaccination rates at 1, 2, 3, and 4 years. At age 2 years, the welfare deduction group had higher vaccine series completion.	(1) The incentives group had higher attendance. (2) No difference between the groups. (3) The welfare deduction group had higher vaccination rates.

^a^RR: relative risk.

^b^I^2^ statistic: percentage of variation due to heterogeneity between studies.

^c^SMD: standardized mean difference.

^d^OR: odds ratio.

### RoB for Included Systematic Reviews

The assessment of the 16 items of AMSTAR-2 from each included review is demonstrated in Multimedia Appendix 3 [22-40]; 11 systematic reviews were rated as critically low [22,23,25,26,30-36] and 8 [24,27-29,37-40] were rated as low quality. Items 7 and 10, as indicated in Table 3, were rated as particularly low quality. All systematic reviews except 1 [29] reported potential sources of conflicts of interest, including any funding they received for conducting the review, but no review reported on the sources of funding for the studies included in the review. It is important to note that AMSTAR-2 does not evaluate the quality of the primary studies. Its objective is to evaluate the methodological quality of the systematic reviews, considering how well the systematic review was conducted (eg, literature searching and data pooling). Therefore, if a systematic review included primary studies with a high RoB but the review itself was well conducted, the review tended to be rated as *high quality*.

In Table 3, we detailed the overall confidence in the results of each included systematic review. Reviews performed poorly with respect to (1) reporting sources of funding for included studies (0/19, 0%), (2) adequately investigating publication bias (small study bias) and discussing its likely impact on the results of the review (4/19, 21%), and (3) providing a list of excluded studies and justifying their exclusions (6/19, 32%).

**Table 3:** Overall confidence assessment (Assessing the Methodological Quality of Systematic Reviews 2 tool) of the 19 included systematic reviews.

AMSTAR-2^a^ items	Yes, n (%)	Partial yes, n (%)	No, n (%)	No MA^b^, n (%)
1. Did the research questions and inclusion criteria for the review include the components of PICO^c^?	13 (68)	0 (0)	6 (32)	0 (0)
2. Did the report of the review contain an explicit statement that the review methods were established before the conduct of the review and did the report justify any significant deviations from the protocol?^d^	7 (37)	8 (42)	4(21)	0 (0)
3. Did the review authors explain their selection of the study designs for inclusion in the review?	12 (63)	0 (0)	7 (37)	0 (0)
4. Did the review authors use a comprehensive literature search strategy?^d^	3 (16)	14 (74)	2 (11)	0 (0)
5. Did the review authors perform study selection in duplicates?	13 (68)	0 (0)	6 (32)	0 (0)
6. Did the review authors perform data extraction in duplicates?	12 (63)	0 (0)	7 (37)	0 (0)
7. Did the review authors provide a list of excluded studies and justify the exclusions?^d^	6 (32)	0 (0)	13 (68)	0 (0)
8. Did the review authors describe the included studies in adequate detail?	13 (68)	3 (16)	3 (16)	0 (0)
9. Did the review authors use a satisfactory technique for assessing the RoB^e^ in individual studies that were included in the review?^d^	9 (47)	6 (32)	4 (21)	0 (0)
10. Did the review authors report on the sources of funding for the studies included in the review?	0 (0)	0 (0)	19 (100)	0 (0)
11. If meta-analysis was performed, did the review authors use appropriate methods for statistical combination of results?^d^	7 (37)	0 (0)	4 (21)	8 (42)
12. If meta-analysis was performed, did the review authors assess the potential impact of RoB in individual studies on the results of the meta-analysis or other evidence synthesis?	6 (32)	0 (0)	5 (26)	8 (42)
13. Did the review authors account for RoB in individual studies when interpreting/discussing the results of the review?^d^	10 (53)	0 (0)	9 (47)	0 (0)
14. Did the review authors provide a satisfactory explanation for and discussion of any heterogeneity observed in the results of the review?	10 (53)	0 (0)	9 (47)	0 (0)
15. If they performed quantitative synthesis, did the review authors carry out an adequate investigation of publication bias (small study bias) and discuss its likely impact on the results of the review?^d^	4 (21)	0 (0)	8 (42)	7 (37)
16. Did the review authors report any potential sources of conflict of interest, including any funding they received for conducting the review?	18 (95)	0 (0)	1 (5)	0 (0)

^a^AMSTAR-2: Assessing the Methodological Quality of Systematic Reviews 2.

^b^MA: meta-analysis.

^c^PICO: population, intervention, control group, outcome.

^d^Items considered as critical domains in the AMSTAR-2.

^e^RoB: risk of bias.

Out of the 19 reviews, 10 (53%) accounted for such bias in individual studies when interpreting and discussing the results of the review and 9 (47%) reviews used a satisfactory technique for assessing the RoB in individual studies included in the review. For reviews that used a satisfactory technique for assessing RoB, most [23,26,27,29,35,36] suggested that e-engagement, participation, and retention promotion strategies were effective.

## Discussion

### Principal Findings

This study reviewed the current state of research comparing alternative strategies to maximize participant engagement, participation, and retention in large digital studies (as held in narrative and systematic reviews). We explored EFIs and study design features that aid engagement, participation, and retention. For reviews that met the inclusion criteria, there was substantial heterogeneity across studies in terms of e-strategies.

Most reviews show that e-engagement and participation promotion strategies are effective, which is promising. However, these reviews canvassed relatively few experimentally tested strategies, suggesting that the myriad of alternative strategies that may have been tested have not yet been the subject of reviews. Many studies have reported these features as a secondary goal of objectives such as adherence to therapy rather than as a primary goal in and of itself. From the 19 reviews, few contained very large digital studies that directly compared alternative strategies to examine impacts on engagement and retention; this may reflect the small number of such mega-studies worldwide. However, contributing reviews contained multipurpose (observational and interventional) cohort studies conducted in clinical and research settings. Motivation for study engagement, participation, and retention may differ somewhat between observational and clinical intervention studies where the participant can potentially directly benefit from participation, although the successful strategies make sense and at face value seem likely to generalize to both settings. In the absence of more tailored evidence, engagement strategies successful in intervention studies may be the best evidence we have, though they should be cautiously applied.

In the context of technical study design features, evidence suggests that using email or SMS text message reminders and voice notifications enhanced participant attendance to health care clinics. Although promising, these results should be interpreted with caution given the short duration of the e-intervention and reliance on self-reported medication adherence measures. Future studies need to determine the features of text message interventions that improve success and appropriate patient populations, sustained effects, and influences on clinical outcomes.

Human facilitation or support was important in influencing the uptake, engagement, and outcomes of digital technologies [26]. As illustrated by completion modules and completer rates, the larger effect sizes found in guided interventions suggested increased intervention adherence.

Reviews examining the effectiveness of e-engagement, participation, and retention interventions in the context of a health care intervention (rather than a cohort study) suggested the following:

1. Visual and personalized feedback seemed effective, for example, for recordings of parent-child interaction in the neonatal intensive care setting [42,43]. This reinforces successes and/or offers motivational support to achieve an individual’s goals and is consistent with known key strategies for behavior change.
2. In e-intervention studies, goal setting has mostly been used as a behavior change strategy [44-46], such as the Fishbein and Yzer Integrative Model of Behavioral Prediction and Fogg Behavior Model for Persuasive Design [44], Theory of Planned Behavior and Fun Theory [47], the Social Cognitive Theory [48,49], and the Coventry, Aberdeen, and London-Refined taxonomy of behavior change techniques [50].
3. Using digital push interventions for vaccine uptake and series completion supported the idea that digital technologies could be a useful adjunct in improving vaccination rates. Reminder interventions for vaccinations have improved the completion of vaccination schedules.
4. There was higher uptake when parental financial incentives or rewards were offered in quasi-mandatory schemes to increase the uptake of preschool vaccinations. Universal gifts were more acceptable than targeted parental financial incentives.
5. Mental health apps were effective or partially effective in producing beneficial changes in psychological outcomes among young adolescents (ie, among college students). This is consistent with past meta-analyses of digital mental health programs for similar populations [51,52].
6. Intensive guidance (with a human coach) was more efficacious than unguided interventions and a beneficial design feature, particularly for mental health studies. It is considered an adherence-facilitating measure in large digital research studies.
7. Electronic text notifications improved attendance and reduced nonattendance (no-shows) across health care settings. Sending multiple notifications improved attendance rates.

Overall, no specific e-intervention strategy was identified as being superior. However, more interactive methods of delivery, such as videos and regular e-therapist contact for training, (1) improve adherence, (2) increase completion rates, and (3) improve fidelity. Further research is needed to understand the strategies that improve retention in longitudinal studies.

### Limitations

We limited our search to systematic reviews published between 2012 and 2019. These reviews should give good reach into source studies during the preceding decade while encompassing the rapid evolution of technology and the explosion of digital methods in this period and thus relevant to the new studies of the 2020s. However, we acknowledge that this is an arbitrary choice. Although all of the literature sourced reported on studies using partial or fully digital contact with participants, much was in the context of interventions and may not be wholly applicable to observational cohort studies. Nonetheless, those strategies found to be successful in interventional settings seem worthwhile to explore in cohort studies. We obtained low-quality ratings for some systematic reviews. We also note that although high engagement and retention are the best strategies to obtain powerful representative data sets, statistical techniques such as multiple imputation are vital adjuncts.

### Conclusions

Although all studies want to maximize the recruitment and retention of study participants, the best methods to do this, particularly in digital settings, are understudied. This review adds to the small but growing literature on methods for optimizing engagement and participation in digital contact cohort studies. Evidence-based recruitment and retention methods are particularly important to the success of the next generation of very large birth cohorts, which are very expensive but have low funding per participant and require high retention throughout decades despite participants having no or very little in-person contact with the study team. Ideally, such studies will not only use existing evidence-based methods but will also build on experimental studies of alternative engagement and retention methods to build the evidence base of *the science of science*.

## Appendix

Multimedia Appendix 1 Search query for systematic reviews and meta-analyses in OVID MEDLINE (search conducted on December 19, 2019).

Multimedia Appendix 2 Study-specific research questions and answers provided in reviews to achieve high participant engagement, participation, and retention.

Multimedia Appendix 3 Methodological quality of included systematic reviews.

## Abbreviations

AMSTAR-2: Assessing the Methodological Quality of Systematic Reviews 2
DI: digital intervention
EFI: engagement-facilitation intervention
GenV: Generation Victoria
NHMRC: National Health and Medical Research Council
PRISMA: Preferred Reporting Items for Systematic Reviews and Meta-Analyses
RoB: risk of bias
