# Supplementary material for: Maximizing Participant Engagement, Participation, and Retention in Cohort Studies Using Digital Methods: Rapid Review to Inform the Next Generation of Very Large Birth Cohorts
Source: J Med Internet Res. 2021 May 14;23(5):e23499. doi: 10.2196/23499 (PMC8164122; doi:10.2196/23499)
Supplement: Multimedia Appendix 1 [file jmir_v23i5e23499_app1.pdf]

Search query for systematic reviews and meta-analyses in OVID Medline (search conducted on December 19, 2019).

**OVID Medline Search Query**
1. patient dropouts/ or patient participation/ or "retention (psychology)"/ or patient compliance/ or compliance/ or "Treatment Adherence and Compliance"/
2. Feasibility Studies/ or "Patient Acceptance of Health Care"/ or Self Efficacy/ or Communication Barriers/
3. Self Care/ or Telemedicine/
4. Electronic Mail/ or Computer Communication Networks/ or Text Messaging/ or internet/ or Social Media/ or Audiovisual Aids/ or software/ or exp mobile applications/ or Reminder Systems/
5. Reward/ or Motivation/
6. Time Factors/
7. (health or disease).tw,kf,hw.
8. 1 or 2
9. 3 or 4 or 5 or 6
10. (((young or emerging) and (adult* or people or person or persons)) or aya or ayas or man or men or woman or women or working-age* or workingage* or middleage* or middle-age* or university-student* or college-student* or TAFE-student* or parent* or mother* or father* or dad or dads or mum or mums).af.
11. 7 and 8 and 9 and 10
12. Limit 11 to (yr=”2012-Current” and english)
13. (systematic adj3 review).ti.
14. meta-analysis.pt.
15. meta-analy*.ti.
16. systematic review.pt.
17. this systematic review.ti,ab,kf,hw.
18. pooling project.ti,ab,kf,hw.
19. systematic review.ti,ab. and review.pt.
20. meta-synthesis.ti.
21. integrative review.ti,ab,kf,hw.
22. integrative research review.ti,ab,kf,hw.
23. (rapid review or umbrella review).ti,ab,kf,hw.
24. consensus development conference.pt.
25. practice guideline.pt.
26. drug class reviews.ti.
27. (cochrane database syst* rev* or acp journal club or health technolog* assess* or evid* rep* technolog* assess* summ* or jbi database system rev* implement rep*).ja,jn.
28. (clinical guideline and management).ti,ab,kf,hw.
29. evidence based.ti.
30. exp Evidence-Based Medicine/
31. best practice*.ti.
32. evidence synthesis.ti,ab.
33. review.pt.
34. exp therapeutics/
35. evaluation studies.pt.
36. validation studies.pt.
37. guideline.pt. or pmcbook.af.
38. (29 or 30 or 31 or 32) and (33 or 34 or 35 or 36 or 37)
39. 13 or 14 or 15 or 16 or 17 or 18 or 19 or 20 or 21 or 22 or 23 or 24 or 25 or 26 or 27 or 28 or 38
40. (Systematic or systematically or study selection).ti,ab,kf,hw. or critical.ti,ab.
41. ((predetermined or inclusion) and criteri*).ti,ab,kf,hw.
42. (exclusion criteri* or main outcome measures or (standard* adj2 care)).ti,ab,kf,hw.
43. 40 or 41 or 42
44. (survey*1 or review*1 or critique).ti,ab. or (overview* or search* or handsearch or appraisal).ti,ab,kf,hw. or Analysis.ti.
45. reduction.ti,ab,kf,hw. and (exp risk/ or risk.ti,ab,kf,hw.) and (exp death/ or exp recurrence/ or (death or recurrence).af.)
46. 44 or 45

47.	(literature or articles or publication* or bibliography or bibliographies or published or database or internet or textbooks or trials or (clinical and studies)).ti,ab. or (pooled data or unpublished or citation* or references or scales or papers or datasets or meta-analy* or treatment outcome).ti,ab,kf,hw. or pmcbook.af.
48.	exp treatment outcome/
49.	47 or 48
50.	(letter or newspaper article or comment).pt. or protocol*.ti.
51.	(39 or (43 and 46 and 49)) not 50
52.	12 and 51

Search query for systematic reviews and meta-analyses in PubMed (search conducted on December 19, 2019).

**PubMed Search Query**
#1 Title/Abstract: retention OR engagement OR completion OR adherence OR participat* OR attrition OR dropout* OR drop-out* OR complian* OR uptake OR enrol* OR feasibility OR acceptability OR acceptance OR efficacy OR barrier* OR recruitment*<br>AND<br>#2 Title/Abstract: self-monitor* OR telemonitor* OR tele-monitor* OR telemedicine OR tele-medicine OR telehealth OR tele-health OR ehealth OR e-health OR mhealth OR mobile-health OR mobilehealth OR digital-health OR email OR emails OR interactive-video* OR interactive-multi-media OR interactive-multimedia OR interactive-technolog* OR electronic OR app OR apps OR online OR text-messag* OR short-message-service* OR sms OR internet OR social-media OR audio-visual-aid* OR audiovisual-aid* OR digital-intervention* OR mobile-application* OR pre-record* OR reminder-system* OR remind* OR prompt* OR feedback OR reward* OR incentive* OR motivat* OR gift OR gifts OR time OR timing OR duration OR “mode of delivery” OR delivery-mode OR interval OR intervals OR frequency<br>AND<br>#3 Title/Abstract: health OR disease<br>AND<br>#4 ((young OR emerging) AND (adult* OR people OR person OR persons)) OR aya OR ayas OR man OR men OR woman OR women OR working-age* OR workingage* OR middleage* OR middle-age* OR university-student* OR college-student* OR TAFE-student* OR parent* OR mother* OR father* OR dad OR dads OR mum OR mums<br>AND<br>*#5 (NOTNLM OR publisher[sb] OR inprocess[sb] OR pubmednotmedline[sb] OR indatareview[sb] OR pubstatusaheadofprint)*<br><br>#6 #1 AND #2 AND #3 AND #4 AND #5<br><br>#7 (((systematic review[ti] OR systematic literature review[ti] OR systematic scoping review[ti] OR systematic narrative review[ti] OR systematic qualitative review[ti] OR systematic evidence review[ti] OR systematic quantitative review[ti] OR systematic meta-review[ti] OR systematic critical review[ti] OR systematic mixed studies review[ti] OR systematic mapping review[ti] OR systematic cochrane review[ti] OR systematic search and review[ti] OR systematic integrative review[ti]) NOT comment[pt] NOT (protocol[ti] OR protocols[ti])) NOT MEDLINE[subset]) OR (Cochrane Database Syst Rev[ta] AND review[pt]) OR systematic review[pt]<br><br>#8 #6 AND #7<br>Limit to: 2012-2020<br>Limit to: English
