# Supplementary material for: Maximizing Participant Engagement, Participation, and Retention in Cohort Studies Using Digital Methods: Rapid Review to Inform the Next Generation of Very Large Birth Cohorts
Source: J Med Internet Res. 2021 May 14;23(5):e23499. doi: 10.2196/23499 (PMC8164122; doi:10.2196/23499)
Supplement: Multimedia Appendix 2 [file jmir_v23i5e23499_app2.pdf]

Study specific research questions and answers provided in reviews to achieve a high participant engagement, participation and retention

Study Research Questions (RQs)	Rapid Review (Yes/No)?	Answers provided in reviews to achieve high participant engagement, participation & retention
RQ1. What technical and study design feature aid engagement, participation and retention?	Yes	Parental financial incentives (including gifts)
		SMS alerts/notifications
		SMS/Email reminders
		Technical feasibility and usability (informed consent)
RQ2. What engagement facilitation interventions (EFIs) aid engagement, participation and retention?	Yes	Interactive voice response messages
		Human guidance/support/facilitation
		Video conferencing and video-feedback interventions (Skype, FaceTime, BabyLink)
		Online modules learning materials or activities,
		Online chat facilities with a professional
		Games and psychoeducational computer programs
		Video training material
RQ3. What feedback is valued by participants with young children?	Yes	Visual and personalised feedback tailored to participant self-monitored data
		Feedback about needs, preferences, and experiences during pregnancy
		Video-feedback
		Feedback from a video interaction guidance
RQ4. How effective are e-Engagement/participation/retention interventions?<br><br>(The effectiveness of each of the e-Strategies explained further below)	Yes	Personalised messages and SMS reminders are more likely to engage participants.
		Using Facebook as a recruitment tool show success in penetrating hard to reach populations as compared with web-based advertising, traditional methods and other social media sites, such as Twitter and MySpace.
		There is higher uptake when parental financial incentives/rewards are offered.
		Reminder interventions for vaccinations could improve vaccination completion
		Mental health apps could be effective, or partially effective, in producing beneficial changes in psychological outcomes in young adolescents
		Intensive guidance is more efficacious than unguided interventions

The Effectiveness of Engagement, Participation and Retention Promoting Strategies
Engagement/Uptake
A meta-analyses by Alkhaldi et al [23] suggested that technology-based strategies could potentially promote engagement compared to no strategy for dichotomous outcomes (relative risk [RR] 1.27, 95% CI 1.01-1.60, I^2 =71%, dichotomous outcomes (n=8)).
Using Facebook as a recruitment tool showed success in penetrating hard to reach populations, finding the results representative of their control or comparison demographic population. Compared with traditional recruitment methods, benefits include reduced costs, shorter recruitment periods, better representation, and improved participant selection in young and hard to reach groups [40]. Adams et al [22] examined the effectiveness and acceptability of parental financial incentives and quasi-mandatory schemes for increasing the uptake of preschool vaccinations. Results suggested higher vaccine uptake when there were rewards or penalties and higher vaccine uptakes for quasi-mandatory programs. Universal gifts were more acceptable than targeted parental financial incentives.

Participation

In the context of child vaccinations, Atkinson et al [25] reported that sending parents SMS's was associated with an 18% increased odds of receiving one/all vaccinations, compared to non-digital contacts such as an appointment card. Parents receiving SMS had an increased odds (OR 1.22 (95%CI, 1.15 - 1.30): 1 dose (n=13)) of participants being vaccinated or completing vaccination series with SMS alerts compared non-digital interventions. Both analyses had high statistical heterogeneity, with I^2 values of 86% and 79% respectively.
In clinical settings, patients who received SMS notifications were 23% more likely to attend healthcare clinics than those who received no notification [36]. Multiple SMS notifications added significantly improved (19% increase) clinic attendance than those who received one notification (Attendance (n=13): RR 1.23 (1.10, 1.38)). Voice notifications offered slight improvements over text notifications for increasing attendance. In a meta-analysis [37], digital reminders (SMS, text message and real time medication monitoring) increased medication adherence, with an approximate doubling of the odds of patients achieving adherence to their medication regimens. This increase translates into adherence rates improving from 50% to 67.8% (or an absolute increase of 17.8%).
Dubad et al [29] considered participants' perceptions of mobile mood-monitoring applications (apps) in young people in terms of acceptability, usability and its clinical impacts. Participation rates ranged from 30% to 99% across studies. Despite positive experiences reported, technological difficulties such as software crashes and reduced battery life were reported to negatively affect user experience and participation. Mertens et al [34] showed telehealth could optimise excessive gestational weight gain (GWG) and postpartum weight retention (PPWR), though not all results were significant. More research is needed to examine the effectiveness, the usability, and the critical features of these interventions.

Completion

Valimaki et al [39] reported in a meta-analyses the effectiveness of Web-based interventions to support adolescents with depression or depressive symptoms, anxiety, and stress. Adolescents in the intervention group left the study early more often, both in short-term studies (Attrition in short-term studies (n =11), P=.007, median 1.31, 95% CI 1.08-1.58) and mid-term studies (P=.02, median 1.65, 95% CI 1.09-2.49). At the end of the intervention, a statistically significant improvement was found in the intervention group regarding depressive symptoms and after 6 months. Anxiety symptoms and moods and feelings improved. There was no difference in stress scores. Lattie et al [32] assessed mental health in college students using digital mental health interventions such as internet-based cognitive behavioural therapy. The number of sessions/assessments/prompts completed, time spent online, dropout rate, participants' perception, usefulness rating for each module, feedback, and participant adherence were outcome measures. Vast majority of included studies reported digital mental health interventions using email reminders (reminder group mean = 2.9, SD = 2.5) as either effective or partially effective in producing beneficial changes in the main psychological outcome variables. Effectiveness did not appear to substantially vary by type of digital mental health intervention, indicating that computer-, web-, mobile-, and virtual reality–based interventions all hold potential for improving mental health on college campuses. There were notable rates of participant attrition and early program discontinuation in many of the studies. In Kang et al's study [31], reminders were also used as a completion strategy for HPV vaccination completion. This study used reminders to encourage users to complete sessions with up to 7 email or SMS reminders as compared to sending paper appointment cards (Number of people completing vaccine schedule (n=86)). The rates of HPV vaccination completeness increased with the interventions. However, the completeness rate of HPV vaccination remained unsatisfactory and lower than its initiation rate.
Belisario et al [27] assessed the impact that smartphone and tablet apps as a delivery mode have on the quality of survey questionnaires. Authors reported that in both controlled and uncontrolled settings, there was no significant differences in the mean overall scores between smart phone applications (apps) and other delivery modes such as paper, laptop computer, tablet computer, SMS and plastic objects. Regarding adherence to the sampling protocol, there were higher completeness in apps than paper (Number of complete responses (n = 21), CI: 2.9 – 11.26)) but no different from SMS. Regardless of study setting however, none of the included studies reported data accuracy or response rates.
