# Supplementary material for: Maximizing Participant Engagement, Participation, and Retention in Cohort Studies Using Digital Methods: Rapid Review to Inform the Next Generation of Very Large Birth Cohorts
Source: J Med Internet Res. 2021 May 14;23(5):e23499. doi: 10.2196/23499 (PMC8164122; doi:10.2196/23499)
Supplement: Multimedia Appendix 3 [file jmir_v23i5e23499_app3.pdf]

Methodological quality of included systematic reviews

Study	AMSTAR-2 Items																Overall rating
	1	2	3	4	5	6	7	8	9	10	11	12	13	14	15	16	
Adams et al. 2015 [22]	Yes	Yes	Yes	Partial Yes	No	Yes	No	Yes	Partial Yes	No	No MA	No MA	Yes	Yes	No	Yes	Critically low
Alkhaldi et al. 2016 [23]	Yes	Yes	Yes	Yes	Yes	Yes	No	Partial Yes	Yes	No	Yes	Yes	No	Yes	No	Yes	Critically low
Ames et al. 2019 [24]	No	Partial Yes	No	Partial Yes	Yes	No	Yes	No	Partial Yes	No	No MA	No MA	No	No	No MA	Yes	Low
Atkinson et al. 2019 [25]	Yes	Partial Yes	No	No	Yes	Yes	No	Yes	Partial Yes	No	Yes	No	No	Yes	Yes	Yes	Critically low
Baumeister et al. 2014 [26]	Yes	Partial Yes	Yes	Yes	No	Yes	No	Partial Yes	Yes	No	Yes	Yes	No	Yes	No	Yes	Critically low
Belisario et al. 2015 [27]	Yes	Partial Yes	Yes	Partial Yes	Yes	Yes	No	Yes	Yes	No	No MA	No MA	Yes	No	No MA	Yes	Low
Dol et al. 2017 [27]	Yes	Yes	Yes	Partial Yes	No	Yes	Yes	Yes	Yes	No	No MA	No MA	No	No	No MA	Yes	Low
Dubad et al. 2017 [29]	No	Partial Yes	Yes	Partial Yes	No	No	No	Partial Yes	Yes	No	No MA	No MA	Yes	No	No MA	No	Low
Garrido et al. 2019 [30]	Yes	No	No	No	Yes	Yes	Yes	No	Partial Yes	No	Yes	Yes	Yes	No	No	Yes	Critically low
Kang et al. 2017 [31]	Yes	Partial yes	No	Partial Yes	No	No	No	Yes	Partial Yes	No	No	No	Yes	Yes	No	Yes	Critically low
Lattie et al. 2019 [32]	Yes	Yes	Yes	Partial Yes	Yes	Yes	No	Yes	Yes	No	No	No	Yes	Yes	No	Yes	Critically low
Lim et al. 2019 [33]	No	Yes	Yes	Yes	Yes	Yes	Yes	Yes	No	No	No MA	No MA	No	No	No MA	Yes	Critically low
Mertens et al. 2019 [34]	Yes	Partial yes	Yes	Partial yes	Yes	Yes	No	Yes	Partial Yes	No	No	No	Yes	Yes	No	Yes	Critically low
Parsons et al. 2017 [35]	Yes	Yes	Yes	Partial Yes	Yes	No	No	Yes	Yes	No	No	No	Yes	No	No	Yes	Critically low
Robotham et al. 2016 [36]	No	No	No	Partial Yes	Yes	Yes	No	Yes	Yes	No	Yes	Yes	Yes	Yes	Yes	Yes	Critically low

Study	AMSTAR-2 Items																Overall rating
	1	2	3	4	5	6	7	8	9	10	11	12	13	14	15	16	
Thakkar et al. 2016 [37]	Yes	Yes	No	Partial Yes	Yes	Yes	Yes	Yes	No	No	Yes	Yes	Yes	Yes	Yes	Yes	Low
Tromp et al. 2015 [38]	No	No	Yes	Partial Yes	Yes	No	No	No	No	No	No MA	No MA	No	No	No MA	Yes	Low
Valimaki et al. 2017 [39]	Yes	Partial Yes	Yes	Partial Yes	Yes	No	Yes	Yes	Yes	No	Yes	Yes	No	Yes	Yes	Yes	Low
Whitaker et al. 2017 [40]	No	No	No	Partial Yes	No	No	No	Yes	No	No	No MA	No MA	No	No	No MA	Yes	Low

Items: 1) Did the research questions and inclusion criteria for the review include the components of PICO?; 2) Did the report of the review contain an explicit statement that the review methods were established prior to the conduct of the review and did the report justify any significant deviations from the protocol?; 3) Did the review authors explain their selection of the study designs for inclusion in the review? 4) Did the review authors use a comprehensive literature search strategy?; 5) Did the review authors perform study selection in duplicate?; 6) Did the review authors perform data extraction in duplicate?; 7) Did the review authors provide a list of excluded studies and justify the exclusions?; 8) Did the review authors describe the included studies in adequate detail?; 9) Did the review authors use a satisfactory technique for assessing the risk of bias (RoB) in individual studies that were included in the review?; 10) Did the review authors report on the sources of funding for the studies included in the review?; 11) If meta-analysis was performed did the review authors use appropriate methods for statistical combination of results?; 12) If meta-analysis was performed, did the review authors assess the potential impact of RoB in individual studies on the results of the meta-analysis or other evidence synthesis?; 13) Did the review authors account for RoB in individual studies when interpreting/ discussing the results of the review?; 14) Did the review authors provide a satisfactory explanation for, and discussion of, any heterogeneity observed in the results of the review?; 15) If they performed quantitative synthesis did the review authors carry out an adequate investigation of publication bias (small study bias) and discuss its likely impact on the results of the review?; 16) Did the review authors report any potential sources of conflict of interest, including any funding they received for conducting the review?
AMSTAR-2, Assessing the Methodological Quality of Systematic Reviews 2; MA, meta-analysis.

Overall confidence of review [19]: 1) *High* (No or one non-critical weakness): the systematic review provides an accurate and comprehensive summary of the results of the available studies that address the question of interest 2) *Moderate* (More than one non-critical weakness*): the systematic review has more than one weakness but no critical flaws. It may provide an accurate summary of the results of the available studies that were included in the review 3) *Low* (One critical flaw with or without non-critical weaknesses): the review has a critical flaw and may not provide an accurate and comprehensive summary of the available studies that address the question of interest 4) *Critically low* (More than one critical flaw with or without non-critical weaknesses): the review has more than one critical flaw and should not be relied on to provide an accurate and comprehensive summary of the available studies
*Multiple non-critical weaknesses may diminish confidence in the review and it may be appropriate to move the overall appraisal down from moderate to low confidence.
Items **2, 4, 7, 9, 11, 13, 15** are considered as critical domains in the AMSTAR-2
